# PRP and PRF—Subgroups and Divisions When Used in Dentistry

**DOI:** 10.3390/jpm11100944

**Published:** 2021-09-23

**Authors:** Paulina Pietruszka, Izabela Chruścicka, Irena Duś-Ilnicka, Anna Paradowska-Stolarz

**Affiliations:** 1 Faculty of Dentistry, Wroclaw Medical University, ul. Krakowska 26, 52-425 Wrocław, Poland; paulina.pietruszka@student.umed.wroc.pl (P.P.); izabelca.chruscicka@student.umed.wroc.pl (I.C.); 2 Department of Oral Pathology, Wroclaw Medical University, ul. Krakowska 26, 52-425 Wrocław, Poland; 3 Department of Dentofacial Anomalies, Department of Orthodontics and Dentofacial Orhopedics, Wroclaw Medical University, Krakowska 26, 52-524 Wrocław, Poland; anna.paradowska-stolarz@umed.wroc.pl

**Keywords:** platelet-rich plasma, platelet-rich fibrin, regenerative endodontics, periodontitis, guided tissue regeneration

## Abstract

Blood derivates, such as platelet-rich plasma (PRP) and platelet-rich fibrin (PRF), are autogenous sources of many growth factors that are involved in the healing and regeneration of tissues, and for this reason, are used in dentistry treatments. This fact also contributes to the growing interest in these biomaterials in regenerative personalized medicine. The multitude of platelet-rich forms creates many possibilities for their use. This semi-systematic review describes and compares the methods of obtaining properties and potential uses of these materials in personalized treatments.

## 1. Introduction to PRP and PRF Subgroup Divisions

The regenerative potential that platelets provide in regenerative medicine was rapidly examined but their usage in oral and maxillofacial surgery was only approved in the late 1990s [1]. Several hypotheses about platelets lie in the understanding of forms of antibacterial action. Platelets play a key role in the immune system [1,2,3]. They secrete molecules that have a defensive effect on the human body. After stimulation by thrombin, platelets release special proteins that have the capacity to inhibit bacteria and fungi activity, e.g., platelet growth factor (PDGF), transforming growth factor (TGF-β) and insulin-like growth factor (IGF-I) [1,4,5]. These factors support biological processes that are necessary for proper healing and regeneration [2,4]. They also produce reactive oxygen species (ROS), bind microorganisms and participate in antibody-dependent cellular cytotoxicity (ADCC) [1,6,7]. Platelets take part in the recognition and neutralization of pathogenic organisms. They also recruit leukocytes to sites of infection and inflammation and modulate their functions [1,4].

Currently, the classification of platelet-rich forms is presented as follows [1,2]:

1. Platelet-rich plasma (PRP):

a) Pure platelet-rich plasma (P-PRP);
b) Leukocyte- and platelet-rich plasma (L-PRP).

2. Platelet-rich fibrin (PRF):

a) Pure platelet-rich fibrin (P-PRF);
b) Leukocyte- and platelet-rich fibrin (L-PRF);
c) Injectable PRF (I-PRF).

In the preparation of PRP, the use of thrombin, calcium or other biologically safe anti-coagulants is often required [8,9,10]. These factors can have an adverse effect on the coagulation process and also lead to an immune response. In the case of PRF, the addition of any additives is not necessarily due to the fibrinogen it contains, which is converted to fibrin under the influence of physiologically available thrombin [3,10,11]. This significantly reduces the risk of postoperative complications [10]. 

The aim of this article was to show the possibilities of using PRP and PRF as a scaffold in the process of tissue regeneration, treatment of intrabony defects and regenerative endodontic treatment. The article was targeted toward dentists of all kinds of specialties, including periodontology, surgery and endodontic/conservative dentistry. For this reason, PubMed, Google Scholar and Mendeley searches were undertaken and the results were skimmed through to look for the articles on this topic. This article lists the methods of obtaining PRP and PRF and their general use in dentistry. As a natural method of treatment, it is desirable to use it for any type of regenerative procedure.

**Figure 1:**
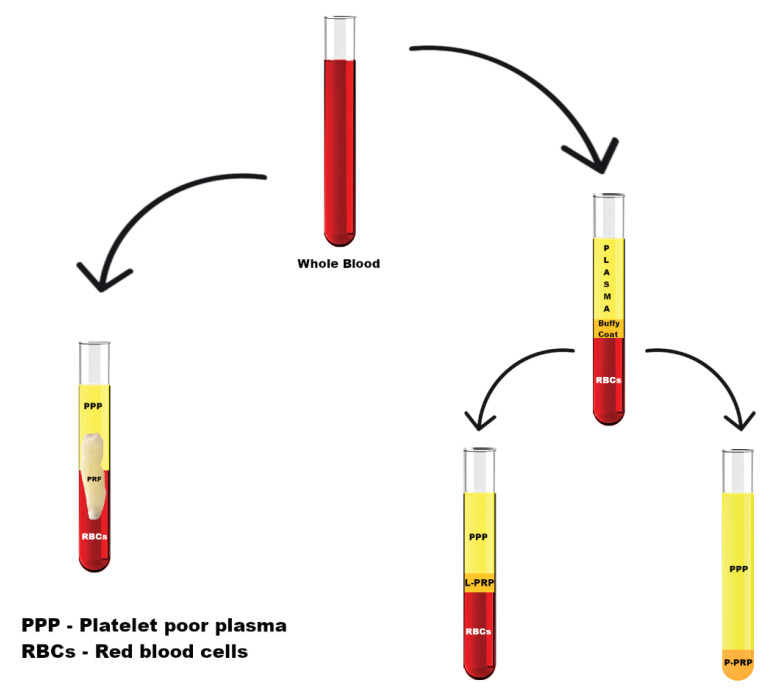
Differences in the methods of obtaining PRF, L-PRP and P-PRP from blood. The left side of the figure reveals the procedure for obtaining PRF, while the right side shows the differences in obtaining P-PRP and L-PRP. PRF: platelet-rich fibrin, P-PRP: Pure platelet-rich plasma, L-PRP: Leukocyte- and platelet-rich plasma.

The differences in the methods of obtaining PRF, L-PRP and P-PRP are presented in Figure 1.

### Molecular Basis of PRP and PRF Methods

Fibroblasts are mesenchymal cells that are responsible for the production of most of the extracellular matrix, which is a very important feature in tissue repair and wound healing [4,12,13]. Platelet-rich plasma (PRP) is widely used in periodontal regeneration [12,14,15].⁠ The primary motivation for using PRP is to promote soft tissue healing [13]. The regenerative effect of PRP occurs because it is dependent on the growth factor pathway, as well as factor-independent pathways [15]. PRP can provide growth factors, for example, platelet growth factor (PDGF) and transforming growth factor beta 2 (TGF-β2) [13]. It is thought that polypeptide growth factors (PGFs) have an important role in the growth and differentiation of cells that are involved in periodontal wound healing [16,17]. They can regulate biological activities, including proliferation, adhesion, migration and cell differentiation, in bone and connective tissue [16]. The use of autogenous platelets is a convenient method that can help to concentrate not only PGF but also epithelial growth factors, vascular endothelial growth factors, insulin-like growth factor (IGF) and basic fibroblast growth factor [13,16]. PDL-derived cells play a key role in periodontal regeneration and their early recruitment is the starting point for regeneration [18,19]. This process can be induced using a mechanism that is dependent on PDGF and/or TGF. Other growth factors included in the PRP, such as basal fibroblast growth factor and epidermal growth factor (EGF), also stimulate PDL cell proliferation [20,21]. However, for this to happen, migration and attachment of connective tissue cells to the wound site and the synthesis of specialized components must also occur [15,18].

Many factors, such as fibrinogen, von Willebrand factor and attachment factors, such as fibronectin, can be released from platelets to form a clot to control bleeding and stabilize the wound [15]. Thus, these components may contribute to the attachment of PDL cells. In addition, PDGF, IGF, EGF and TGF-β were found to stimulate cell migration [12,22].

## 2. Materials and Methods

This review used publications that were searched for through the PubMed, Google Scholar and Mendeley databases. After the first search on those medical platforms, the articles’ abstracts were read to confirm their correlation with the review’s aim. The most interesting and up-to-date articles from the patient’s perspective were taken into account, with most interest given to articles that were published up to 10 years ago. The articles without clear scientific background were rejected.

## 3. Leucocyte Platelet-Rich Fibrin (L-PRF)

L-PRF and the associated blood clot are more homogeneous and fibrous compared with the gelatinous L-PRP. It comes in the form of a thick gel or liquid that can therefore be used in narrow spaces, where it can simply and easily adapt [23]. It contains several factors that accelerate angiogenesis, chemotaxis, mitosis and cell proliferation [23,24]. The results of the related research indicated a beneficial antimicrobial effect of L-PRF and the release of growth factors and matrix proteins for over 7 days [24,25].

The effect of L-PRF was compared with other platelet-rich preparations. Activities against *P. gingivalis*, *A. actinomycetemcomitants* and plaque microorganisms were studied. Studies yielded mixed results. In the study undertaken by Badade et al. [26], no antibacterial effect of L-PRF was detected, unlike PRP, which showed such an effect. The results deviating from the study mentioned above were obtained in 2019 by Karde et al. [27], where the antibacterial activity of both PRF and PRP was observed; however, the zone of inhibition was significantly larger when PRP was used. 

## 4. Use of Platelet-Rich Plasma in Personalized Dentistry

Depending on the method of preparation, PRP containing eight times more thrombocytes than in the initial blood can be obtained. Double centrifugation enables one to obtain a higher concentration of thrombocytes and leukocytes in comparison with single centrifugation [28]. We can distinguish platelet-rich plasma containing only thrombocytes, which is referred to as pure PRP (P-PRP), and platelet-rich plasma with the addition of leukocytes (L-PRP) [1]. They can be used as an injectable suspension or fibrin gels [4]. Compared to i-PRF and L-PRF, P-PRP releases most of the growth factors in the first hours and dissolves completely after 3 days [24]. L-PRP also releases most of the growth factors in the first couple of hours after its inception [29].

### 4.1. Description of Pure Platelet-Rich Plasma (P-PRP) Use in Dentistry

For years, scientists have been trying to understand PRP’s activity against various microorganisms. Bielecki et al. [30] showed the antibacterial effect of P-PRP against microorganisms, such as *Enterococcus faecalis, Candida albicans, Streptococcus agalactiae* and *Streptococcus oralis*. In this research, activity against *P. aeruginosa* was not confirmed. In the study conducted by Yang et al. [31], the activity of P-PRP against many microorganisms was checked, for example, *F. nucleatum*, *P. gingivalis*, *A. actynomicetemcomitans*, *E. coli* and *K pneumoniae*. Antibacterial activity was shown for all these forms. Prabhdeep et al. [10] compared the activity of PRP, platelet-poor plasma, platelet-depleted plasma and PRF relative to *P. gingivalis* and *A. actinomycetemcomitans*. Observations showed the highest PRP activity on the microorganisms mentioned above.

Research containing similar results to Yang et al. [31] was published in 2019 in Vietnam. Pham et al. [32] studied the antimicrobial effect of PRP against *P. gingivalis*, which is one of the main pathogens that are responsible for periodontitis. The main difference was the use of 12.5% PRP by Pham et al. [32] in relation to the 50% PRP that was used by Yang et al. [31]. In Vietnam, healthy patients and patients with periodontitis showed a reduction in *P. gingivalis* adhesion and a significant reduction in bacterial counts in samples that were treated with PRP. In a study conducted by Mariani et al. [2], growth was inhibited for up to 2 h, but later bacterial regrowth was observed.

### 4.2. Use of Leucocyte Platelet-Rich Plasma (L-PRP) in Dentistry

In the study conducted by Ameer et al. [33], the anti-inflammatory effect of PRP was investigated in the periodontal pocket. There was a reduction in all clinical periodontal parameters and the number of noticeable lymphocytes, which are responsible for inflammation and destruction of periodontal tissues. 

Two independent studies that were conducted by Bielecki et al. [30] and Moojen et al. [34] showed antibacterial activity against *S. aureus* and *E. coli*, while no activity was observed for *K. pneumoniae*, *E. faecalis* and *P. aeruginosa*. In these studies activated by bovine thrombin L-PRP was used. It was observed that all platelet concentrates tended to inhibit the growth of microorganisms only during the first few hours of incubation [2,35,36]. Over time, the colonies recovered, which indicates that platelet concentrates demonstrate bacteriostatic rather than bactericidal activity [31,37].

## 5. Injectable Platelet-Rich Fibrin (I-PRF) and Personalized Dental Procedures

The concept of using I-PRF is similar to PRF; however, I-PRF is available as an injection [24,38,39]. It can be used alone or together with other biomaterials [10]. No additives are required to produce I-PRF [27]. I-PRF forms a small clot due to the presence of fibrin [10,24]. These clots behave like dynamic gel-containing cells and release additional growth factors [24,39,40]. It is believed that I-PRF also contains stem cells and endothelial cells [27].

In 2017, Karde et al. [27] observed the largest bacterial growth inhibition zone among plaque microorganisms when I-PRF was used. It was smaller for PRP and the smallest when PRF was used. Prabhdeep et al. [10] indicated the antibacterial activity of PRF, PRP and I-PRF. I-PRF showed the strongest action against *Porphyromonas gingivalis.* Additionally, PRP showed greater activity than PRF. In the case of *Aggregatibacter actinomycetemcomitans*, PRP showed a significantly larger inhibitory zone compared to I-PRF and PRF.

As for today, the results of research conducted around the world do not provide an understanding of the antimicrobial effect of platelet concentrates. Therefore, it is worth perceiving platelets as both an antimicrobial and regenerative agent, which increases the chance of effective therapy [1,10,33].

## 6. Regenerative Treatments with the Use of PRP and PRF in Dentistry

### 6.1. PRP in Gingival Fibroblast Proliferation—Tissue Regeneration

In the first generation of platelet gels used in the therapy of periodontal regeneration, approximately 3–5 times more platelets were used than their normal level in the body [14,16,37]. Nguyen and Pham [13] claimed that due to the high concentration of platelets in PRP, the number of available growth factors (in wound tissue) that could activate cells increases.

In addition to growth factors, PRP can increase the type I collagen expression by periodontal ligament (PDL) cells [16,38]. Recent studies showed that PRP can stimulate the proliferation of PDL cells [16,39,40]. Research conducted by Rattanasuwan et al. [15] confirmed that PRP can modulate the proliferation, attachment and migration of PDL cells in vitro. It was found that the number of cells after PRP treatment (group II—5% PRP and III—10% PRP) was significantly higher than in the group without PRP (group I), but no statistical difference was found between those groups. In addition, the number of attached cells in the groups in which PRP was used was higher than in the group without PRP [15]. This study showed that the number of PDL fibroblast cells attached to dentin plates was significantly increased in the 10% PRP group compared to the 5% PRP group and the control group [15]. Nguyen and Pham [13] also claimed that it can be concluded that in vitro human gingival fibroblast (hGF) traits depend on the concentration of PRP in the medium. So far, some recent studies have shown that increasing the levels of PRP leads to increased proliferation in cells [9,10].

Much of the research done in the recent past focused on the low concentration of platelets in PRP [14,38,41]. Tavassoli-Hojjati et al. [42] showed that media with 0.1 or 5% PRP content were much more effective than media with 50% PRP. Nguyen and Pham [13] claimed these results almost showed that PRP at high concentration had worse effectiveness than at low concentration. Wang et al. [38] found that gingival fibroblasts proliferated better at 1 and 5% PRP compared to those with 10 and 20% PRP. Graziani et al. [12] found that higher concentrations of PRP can reduce cell proliferation. The cell proliferation test conducted by Nguyen and Pham [13] showed that the cell number threshold was reached on the third day (for the 2 and 5% PRP groups) and on the fifth day (for the 1% PRP group). Similar relationships were noted by Creeper et al. [43]. It was concluded that different concentrations of PRP do not promote DNA synthesis in the short term (24 h), while in the long term (5 days), they stimulate the growth of cell proliferation [14,44,45]. 

In contrast, in a study conducted by Hsu et al. [46] and Okuda et al. [47], it was found that treatment with 0.5–5% PRP stimulated the proliferation of PDL cells and osteoblasts. However, Hsu’s research confirmed that the number of cells decreased at high PRP concentrations (15–30%) [46]. Creeper et al. [48] observed a cytotoxic effect at 100% PRP.

### 6.2. Regenerative Endodontic Treatment (RET) and Blood Derivatives

The revascularization process gained popularity in 2004 when Banchs and Trope achieved success in treating immature permanent or nonvital teeth with this method [49]. RET is a modern method of treating immature permanent teeth with necrotic pulp, where such teeth were traditionally been treated using apexification [11]. The RET method is the most effective when no more than two-thirds of the root is created in a tooth with an open apex [49,50]. Teeth in which the root formation process is almost complete show a similar response to RET treatment and the use of MTA [49]. Table 1 summarizes the data on PRF, PRP and BC (blood clot) effects in endodontic treatments.

Regenerative endodontic treatment is based on the concept of tissue engineering and aims to create favorable conditions for the migration, proliferation and differentiation of stem cells [5,11,55]. In this method, the dentist passes an endodontic tool through the tooth’s apical foramen, which causes bleeding and, consequently, the formation of a blood clot under the cemental enamel junction. This technique is effective, but it has many disadvantages; therefore, agents with fewer side effects have been sought [11,56].

Research conducted in recent years showed the potential of blood concentrates as a scaffold in the process of tissue regeneration [51,52,53]. Numerous scientists compared the effectiveness of PRF, PRP and the induced bleeding technique. Narang et al. [51] concluded that PRF has much more potential regarding the tissue regeneration process compared to PRP and blood clots. The data collected by Murray [53] suggested similar effectiveness of the methods mentioned above for the treatment of periapical lesion, root elongation and increase of dental wall thickness. A significant advantage of PRF and PRP over BCR was found in the aspect of apical closure. Compared with the authors mentioned above, Shivashankar et al. [52] suggested that PRP exhibited better activity than PRF and the induced bleeding technique in relation to periapical wound healing when used in regenerative endodontic procedures.

The results obtained by Alagl et al. [14] in a study conducted on 32 immature teeth with apical periodontitis or abscesses characterized by a negative pulp reaction indicated the effectiveness of using PRP as a scaffold in the RET method. Computed tomography showed that the areas of lesions subsided or decreased and bone density increased in all teeth. Despite the rapid action of PRP, the authors drew attention to the disadvantages that are associated with this method, including the need for the taking of blood from the patient [25,52,53]. Because of this, they recommend the technique of induced bleeding as a standard procedure for the revascularization of non-vital immature permanent teeth, which gives delayed results but is less onerous [52,53]. The data obtained by Lv et al. [11] did not show a significant difference between the use of PRF and the technique of inducing periapical bleeding in tooth revascularization or revitalization. On the other hand, the study conducted by Sharma et al. [54] indicated better revascularization properties of PRF and collagen compared to blood clots and commercially available scaffolds, such as collagen and poly(lactic-co-glycolic acid) (PLGA), in immature permanent teeth.

The use of PRF in the form of a membrane is easier and the whole procedure is faster than in the case of a gel. Authors stated that the use of PRF in both forms, namely, membrane and gel, can be used with similar effectiveness [40,57,58]. 

Periodontitis is the breakdown of periodontal tissues, which occurs along with bone loss and clinical attachment, as well as apical migration of the junctional epithelium, which can induce the formation of vertical (angular) and/or horizontal bone defects [25,26]. It manifests in the inflammatory response of periodontal tissues to periodontal pathogens [28,57]. Risk factors for this disease are poor oral hygiene, smoking, genetic predisposition and many systemic diseases, such as diabetes, cardiovascular disease and stroke [25,27].

### 6.3. PRP and PRF in Intrabony Defect Treatment

The main goal of periodontitis therapy is not only to slow down the progression of the disease but also to restore the original function and shape of the periodontal complex [54,59].⁠ The starting point in the treatment of periodontitis is the motivation for oral hygiene and non-surgical therapy [28,57]. First-line therapy is an elimination treatment, e.g., scaling and root planning (SRP) [28,58,60]. However, this has some limitation, e.g., does not remove pathogens from deep intraosseous defects, which can lead to the spread of infection; it can excessively remove tooth tissue; it can cause the formation of a smear layer that hinders periodontal regeneration; and it makes it difficult to access anatomical features, such as root concavities and furcations [58,61,62].

Regenerative therapies, such as enamel matrix derivative product application, guided tissue regeneration, bone grafting or biomolecular techniques, are also used [57,63,64]. For this purpose, many different materials are used, including demineralized freeze-dried bone allografts (DFDBA), autogenic bone, alloplastic materials or xenogenic materials, such as anorganic bovine bone mineral (ABBM), which is a material that is produced by removing organic components from cortical or spongy bovine bone [54,57,60]. Their goal is to obtain a new periodontal connection with new cement, periodontal ligament and alveolar bone. Regrettably, these solutions do not fully restore the complex periodontal structure, which includes alveolar bone, periodontal ligament tissue (PDL) and cementum [57,60].

Shukla et al. [59] claimed that the gold standard in treatment using graft materials is autografting; however, it can lead to complications, such as ankylosis and root resorption. It was investigated that graft materials provide adequate conditions for the ingrowth of osteogenic elements [54,65]. They act osteoconductively, creating a mechanical scaffold to create a new bone [45,65].

In recent years, interest has focused on the field of biological mediators that can improve wound healing and enhance the benefits of bone grafts [58,66,67]. The use of biological mediators can selectively increase the cellular renewal of periodontal wounds [65,67,68]. Thus, the combination of scaffold materials and biologically active agents can have a positive effect on the treatment of intrabony defects by promoting periodontal regeneration because this combination can give synergistic effects [54,65]. Graft materials may act as an osteoconductive scaffold, while bioactive materials can induce the formation of periodontal ligaments, root cement and bones [54,65,66].

One of the biomaterials is an autologous platelet concentrate that aims to provide high levels of polypeptide growth factor in periodontal wounds. PRF acts as a fibrin glue due to its mechanical adhesive properties and biological functions [65,68]. ⁠Compressed PRF can be placed in a defect or used to cover a defect like a guided tissue regeneration (GTR) membrane, facilitating the development of vascularization and directing the migration of epithelial cells to its surface [54,65,69].

The usefulness of autologous PRF in the treatment of defects inside human bones was investigated in several experiments [54,60,65,68]. In one of them, conducted by Sezgin et al. [68], for the test group, PRF was mixed with ABBM granules, the mixture was added to the defect and another portion of PRF was used to cover the defect. For the control group, ABBM alone was prepared and placed in the defect. In summary, only the CAL increase was statistically significant in the test group compared to the control group. Other parameters showed improvement over time but did not show significant differences between the groups [68].

The purpose of the experiment carried out by Zhou et al. [62] was to compare the effectiveness of four types of biomaterials: PRP, PRF, enamel matrix derivative (EMD) and amnion membrane (AM), in combination with demineralized freeze-dried bone allografts (DFDBA). It was found that all subgroups that used PRP and PRF showed statistically significant differences compared to DFDBA alone: both PRP and PRF were shown to have a positive effect on CAL and PD. However, the use of PRF led to better CAL enhancement and PD reduction than PRP. The authors suggested that PRF promoted soft tissue healing, while PRP had a greater impact on hard tissue reconstruction [62].

The results of studies on the improvement of periodontal regeneration after the addition of biomaterials are contradictory. Some of them [54,69] suggest that regeneration using autologous platelet concentrates is more effective than using graft materials alone because graft materials can act as an osteoconductive scaffold [54,60,65], while bioactive materials can induce periodontal tissue formation [62,70], whereas some of the research [68,71,72] does not show any significant improvement in the therapy of intrabony defects with the use of biomaterials compared to the therapy with scaffold materials alone.

## 7. Other Use

Regenerative dentistry is not the only reason to use PRF in dentistry. It is also very useful in the treatment of medical conditions related to oral cavity mucosa or prevention and the treatment of osteonecrosis [73,74]. There is some evidence that PRF might be useful in the treatment (extension) of oral lichen planus (OLP) and symptomatology, although further studies on this topic are required [73]. The application of platelet concentrates in the prevention and treatment of osteonecrosis is still disputable. The result of using them was not clarified and its effectiveness has not been clearly shown [74].

## 8. Conclusions and Perspectives

The results of the research that has been carried out so far are promising, but there is a need for further research in the field of PRP and PRF use in personalized dentistry. PRF and PRP are natural products; therefore, they are biodegradable and biocompatible. For this reason, there is no unneeded waste while obtaining them. Due to their biocompatibility, they cause no allergic reactions. For this reason, platelet concentrates are very interesting for multiple uses in medicine, including dentistry. These kinds of properties are now the most desirable ones, as the products that cause no allergies would be accepted by any organism. This makes PRP and PRF universal products for the treatment of many conditions. The multitude of platelet-rich forms creates many possibilities for their use, for example, in tissue regeneration, intrabony treatments or regenerative endodontic treatments. 

## Figures and Tables

**Table 1:** Description of the results observed by Lv et al. [11], Narang et al. [51], Shivashankar et al. [52], Murray [53] and Sharma et al. [54].

Authors	Substance/Division into Groups	Effects
Lv et al. [11]	PRF	No significant difference between PRF and the technique of inducing bleeding.
Narang et al. [51]	Blood clot (group II) PRF (group III) PRP transferred to collagen (group IV)	Periapical healing No statistically significant difference between groups II and IV; the best result was found for group III (98%). Apical closure No statistically significant difference between groups II–IV. Root lengthening No statistically significant difference between groups II and IV; the best result was found for group II (99%). Dental wall thickness No statistically significant difference between groups II and IV despite the better result in group 2; the best result was found for group III (60%).
Shivashankar et al. [52]	PRF (group A) Induced bleeding (group B) PRP (group C)	After 3 months: No statistically significant difference between groups A–C. After 12 months: The best results in group C.
Murray [53]	PRP PRF BCR (blood clot)	After 12 months: Periapical lesion No statistically significant difference between the groups. Apical closure Higher effectiveness of PRP and PRF than BCR. Root lengthening No statistically significant difference between the groups. Dental wall thickness No statistically significant difference between the groups.
Sharma et al. [54]	Blood clot (group I) PRF (group II) Collagen (group III) PLGA (group IV)	Periapical healing Statistically significant difference between groups II–IV; the best result was found for group II (75%), while the worst result was for group IV. Apical closure, root lengthening, dentinal wall thickening No statistically significant difference between the groups.

## Data Availability

No additional data available.
